# Donor antigen-primed regulatory T cells permit liver regeneration and phenotype correction in hemophilia A mouse by allogeneic bone marrow stem cells

**DOI:** 10.1186/s13287-015-0119-9

**Published:** 2015-07-08

**Authors:** Veena Kochat, Sumod Kanjirakkuzhiyil, Prakash Baligar, Perumal Nagarajan, Asok Mukhopadhyay

**Affiliations:** Stem Cell Biology Laboratory, National Institute of Immunology, Aruna Asaf Ali Marg, New Delhi, 110067 India; Experimental Animal Facility, National Institute of Immunology, New Delhi, India

## Abstract

**Introduction:**

Cell replacement therapy may be considered as an alternate approach to provide therapeutic dose of plasma factor VIII (FVIII) in patients with hemophilia A (HA). However, immune rejection limits the use of allogeneic cells in this mode of therapy. Here, we have examined the role of donor major histocompatibility complex (MHC)-stimulated host CD4^+^CD25^+^ regulatory T (T_reg_) cells in suppressing immune responses against allogeneic uncommitted (Lin^−^) bone marrow cells (BMCs) for correction of bleeding disorder in HA mice.

**Methods:**

Allogeneic donor Lin^−^ BMCs were co-transplanted with allo-antigen sensitized T_reg_ cells in HA mice having acetaminophen-induced acute liver injury. Plasma FVIII activity was determined by *in vitro* functional assay, and correction of bleeding phenotype was assessed on the basis of capillary blood clotting time and tail-clip challenge. The immunosuppression potential of the sensitized T_reg_ cells on CD4^+^ T cells was studied both *in vitro* and *in vivo*. Suppression of inflammatory reactions in the liver against the homed donor cells by sensitized T_reg_ cells was analysed by histopathological scoring. Allo-specificity of sensitized T_reg_ cells and long-term retention of immunosuppression were examined against a third-party donor and by secondary challenge of allogeneic donor cells, respectively. The engraftment and phenotype change of donor BMCs in the liver and their role in synthesis of FVIII and liver regeneration were also determined.

**Results:**

Co-transplantation of allogeneic Lin^−^ BMCs with sensitized T_reg_ cells led to systemic immune modulation and suppression of inflammatory reactions in the liver, allowing better engraftment of allogeneic cells in the liver. Allo-antigen priming led to allo-specific immune suppression even after 1 year of transplantation. Donor-derived endothelial cells expressed FVIII in HA mice, leading to the correction of bleeding phenotype. Donor-derived hepatocyte-like cells, which constitute the major fraction of engrafted cells, supported regeneration of the liver after acute injury.

**Conclusions:**

A highly proficient FVIII secreting core system can be created in regenerating liver by transplanting allogeneic Lin^−^ BMCs in HA mice where transplantation tolerance against donor antigens can be induced by *in vitro* allo-antigen primed T_reg_ cells. This strategy can be beneficial in treatment of genetic liver disorders for achieving prophylactic levels of the missing proteins.

**Electronic supplementary material:**

The online version of this article (doi:10.1186/s13287-015-0119-9) contains supplementary material, which is available to authorized users.

## Introduction

Hemophilia A (HA) is an X-linked autosomal recessive bleeding disorder in which factor VIII (FVIII) is inadequately synthesized. In humans, FVIII is found to be synthesized by liver sinusoidal endothelial cells (LSECs) [1]. Gene knockout studies have recently confirmed that endothelial cells (ECs) are the principal source of plasma FVIII [2, 3]. In treatment of HA, gene replacement therapy showed initially encouraging results in life-long correction of HA in animal models [4–6], although the outcome of the phase I clinical trial was not conclusive; there was a gradual loss of its potency because of the formation of inhibitors [7]. As an alternative to gene therapy, transplantation of LSECs has shown encouraging therapeutic benefits in HA mice [8]. Owing to a profound shortage of transplantable donor LSECs, bone marrow cell (BMC) therapy is considered as an alternative for these patients. Attempts have been made to correct some genetic liver diseases by transplanting BMCs, which are capable of engrafting in the liver and replacing the parenchyma in the regenerating liver micro-environment and thereby produce prophylactic levels of missing proteins [9–12]. All of the above studies were based on transplantation of syngeneic BM-derived cells in mice with perturbed liver in which no humoral response to the missing proteins was observed.

Owing to intrinsic genetic defects, autologous cells cannot be used for therapeutic correction of HA. Immunosuppressants can be used to avoid rejection of donor cells but have serious side effects on long-term administration. CD4^+^ T cells of the recipient act as a double-edged sword; they play a central role in rejection of allograft and are also involved in developing peripheral tolerance against the effector T cells. A subpopulation of CD4^+^ T cells, known as regulatory T (T_reg_) cells, possesses immuno-modulatory properties that are capable of establishing transplant tolerance [13]. Thus, T_reg_ cells are considered a good candidate to overcome the rejection of allogeneic donor cells.

In this report, we have developed allo-antigen-specific T_reg_ cells of recipient background, which can improve the therapeutic benefit of allogeneic Lin^−^ BMCs in HA mice. This strategy facilitates allo-specific immunosuppression, establishes transplant tolerance, and allows better engraftment of donor cells in the regenerating liver. The donor-derived cells helped in regeneration of the liver as well as in synthesis of FVIII protein that led to bleeding phenotype correction in HA mice.

## Methods

### Animals

Six- to eight-week-old HA mice [B6;129S4-F8^tm1Kaz^/J], C57Bl6/J, enhanced green fluorescence protein (eGFP)-expressing Bl6/J [C57Bl6/J-Tg(UBCGFP) 30Scha/J], FVB/J, eGFP-expressing FVB/J [FVB.Cg-Tg(CAGEGFP)B5Nagy/J], and Balb/c mice were used in this study. Mice were obtained from The Jackson Laboratory (Bar Harbor, ME, USA) and maintained in individually ventilated cages and fed with autoclaved acidified water and irradiated food *ad libitum* in the experimental animal facility of the institute. All experiments were conducted in accordance with procedures approved by the Institutional Animal Ethics Committee at the National Institute of Immunology.

### Flow cytometry

Single-cell suspensions of BM, spleen, and liver were prepared [14, 15]. Antibody staining of cells was performed at 4 °C for 30 min. For biotinylated primary antibodies, the washed cells were further stained with fluorochrome-conjugated streptavidin or secondary antibodies. Cells were washed in phosphate-buffered saline-bovine serum albumin (PBS-BSA) buffer and subjected to either analysis or sorting (FACS AriaIII; BD Pharmingen, San Diego, CA, USA). The antibodies and conjugates used for the study were anti-CD4/biotin, anti-CD25/PE, anti-Foxp3/AF647, Streptavidin/PerCP, and Streptavidin/APCCy7 (all from BD Pharmingen); anti-CD11c/PE and anti-CD44/eFluor 450 (both from eBioscience, San Diego, CA, USA); and anti-CD31/biotin (BioLegend, San Diego, CA, USA).

### Donor antigen sensitized T_reg_ cells and characterization

CD4^+^CD25^+^ T_reg_ (nT_reg_) cells of HA mouse spleen were co-cultured with equal number of irradiated (1200 cGy) dendritic cells (DCs) of FVB/J mouse for 48 h. The suppressive effect of T_reg_ cells on proliferation of CD4^+^ T cells was determined by carboxyfluorescein succinimidyl ester (CFSE) (Vybrant^®^ CFDA Cell Tracer kit; Invitrogen, Carlsbad, CA, USA) dilution assay, and interleukin-10 (IL-10) secretion was estimated by using enzyme-linked immunosorbent assay (eBioscience). In T-cell suppression assay, CD4^+^CD25^−^ T cells were labeled with 5 μM CFSE by incubating for 3 min at 37 °C. DCs from FVB/J mice (1 × 10^5^ cells) and CFSE-labeled CD4^+^CD25^−^ T cells from HA mice (1 × 10^5^ cells) were taken in each well of 96-well round-bottom plate in triplicate. The nT_reg_ or sT_reg_ cells were added in each well at different ratios to T cells (0, 0.01, 0.02, 0.04, 0.1, 0.25, 0.5, and 1) and cultured for 5 days. The dilutions of dye in viable CFSE-labeled cells were determined by flowcytometric analysis. Cellular viability was checked with 9 nM Sytox Red (S34859; Molecular Probes Inc., Eugene, OR, USA).

### Transplantation of cells and phenotypic correction

BM progenitor cells (Lin^−^ BMCs) were isolated from eGFP-expressing FVB/J and Balb/c female mice following negative selection with MACS (Miltenyi Biotec, Gladbach, Germany). Acute liver injury was induced by administering acetaminophen (500 mg/kg body weight) in male HA mice through intra-peritoneal injection. Within 30 h of liver injury, 0.25 × 10^6^ Lin^−^ BMCs (eGFP) without or with 0.25 × 10^5^ sT_reg_/nT_reg_ cells were transplanted through tail vein injection. FVIII activity in plasma was measured by using a Coatest SP4 FVIII kit (Chromogenix; Instrumentation Laboratory SpA, Milan, Italy). Correction of bleeding phenotype was assessed by the tail-clip challenge test and whole blood clotting time. In the tail-clip challenge test, tails were clipped at a length of 1.5 cm without subsequent cauterization. Mice were observed for 24 h, and percentage survival was noted. The whole blood clotting time was determined by drawing blood into a capillary tube from the retro-orbital plexus. The loaded capillary tube was broken at regular intervals starting from one end and slowly pulled apart to observe for fine strands of insoluble fibrin. Time taken to produce the visible clot was recorded.

### Liver histopathology

Liver tissues were fixed in 10 % buffered formalin. Paraffin sections were stained with hematoxylin and eosin. Histopathological abnormalities in the liver tissue sections were scored in accordance with a published protocol [16]. Histopathological scoring of inflammatory reactions are included in Additional file 1: Table S1.

### Immunostaining of cells/tissues

Four percent paraformaldehyde-fixed cells were cytospined on poly-L-Lysine-coated glass slides. Liver tissue was frozen in tissue freezing medium (Jung; Leica Microsystems, Wetzlar, Germany), and 5-μm sections were cut. The cells or tissue sections were permeabilised with 0.1 % saponin in PBS for 30 min and then blocked with 3 % BSA solution for 1 h. Samples were stained with primary antibodies overnight at 4 °C, washed, and further stained with Alexafluor-conjugated secondary antibodies (Molecular Probes Inc.) at room temperature for 1 h. Washed cells/sections were briefly treated with 4′,6-diamidino-2-phenylindole (DAPI) (10 μg/ml) and mounted on glass slides by using ProLongR anti-fade (Molecular Probes Inc.). Antibodies used for immunostaining were anti-GFP (Clontech-Takara Bio Company, Kyoto, Japan), anti-CD31 biotinylated (BioLegend) anti-FVIII(lc) (Santa Cruz Biotechnology Inc., Dallas, TX, USA), anti-albumin (Bethyl Laboratories Montgomery, TX, USA), and anti-Foxp3 and anti-F4/80 (eBioscience). Foxp3 staining was performed following DAB-HRP (3, 3′-diaminobenzidine-horseradish peroxidase) method (Vector Laboratories, Burlingame, CA, USA). The stained sections or cells were observed under an Olympus fluorescence microscope, and images were acquired by DP70 digital camera (Model IX51; Olympus, Tokyo, Japan) using LCPlanFl 20× and 60× objectives and Image Pro software (Media Cybernetics, Inc., Rockville, MD, USA). High-magnification images were taken with a Zeiss LSM 510 META confocal laser-scanning microscope (Carl Zeiss, Oberkochen, Germany) using a Plan-Apochromat 63×/1.4 oil objective with LSM 510 software for image acquisition and Zeiss LSM Image browser version 4.2.0.121 for processing. Relative cell fluorescence was measured by using ImageJ software (National Institutes of Health, Bethesda, MD, USA).

### Low-density lipoprotein uptake assay

Low-density lipoprotein (LDL) uptake by ECs was assessed by fluorescence microscopy after incubation of the cells with 10 μg/ml acetylated LDL labeled with 1,19-dioctadecyl-3,3,39,39-tetramethylindo-carbocyanine perchlorate (DiI-Ac-LDL) (Molecular Probes Inc.) for 2 h at 37 °C.

### Gene expression

Total RNA was isolated from purified cellular fractions by using TRI Reagent (Invitrogen), and cDNA was synthesized by using a High Capacity cDNA Reverse Transcription Kit (Applied Biosystems, part of Thermo Fisher Scientific Inc., Waltham, MA, USA). Real-time PCR was performed by using SYBR green technology (Applied Biosystems) in a Stratagene Mx3000P QPCR System (Agilent, Santa Clara, CA, USA). The primers used for the PCR are *Gapdh* (forward primer: 5′-ACGGCCGCATCTTCTTGTGCA-3′, reverse primer: 5′-CAGGCGCCCAATACGGCCAA-3′) and *FVIII* (forward primer: 5′-GCCTGGGCTTA TTTCTC TGATG-3′, reverse primer: 5′-TGAGCAGGATTCAGTGTGTTCG-3′).

### Statistics

Results of multiple experiments were reported as mean ± standard error of the mean. Student’s *t* test was carried out to calculate the significance between the means of both groups, and a *P* value of less than 0.05 was considered significant. All analyses were carried out by using GraphPad Prism software, version 5.02 (GraphPad Software, Inc., La Jolla, CA, USA).

## Results

### Allogeneic Lin^−^ BMCs protect HA mice from bleeding challenge in the presence of sT_reg_ cells

**Fig. 1:**
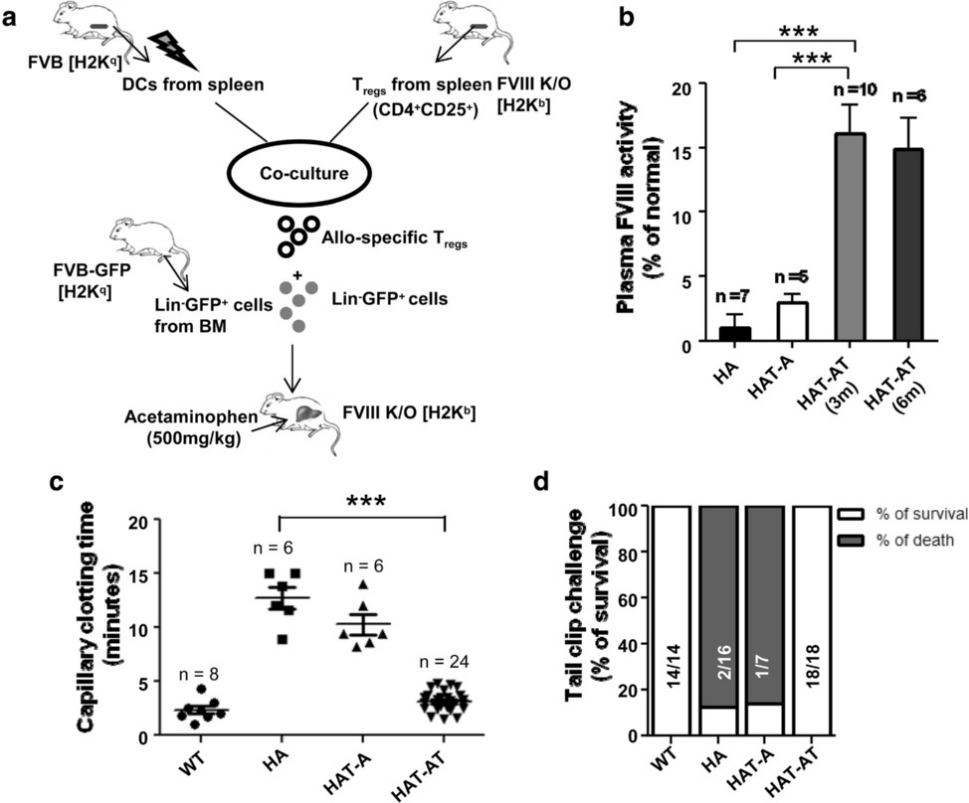
Co-transplantation of sT_reg_ cells and Lin^−^ BMCs facilitates therapeutic correction in HA mice. **a** Scheme of the experiment. Lin^−^ BMCs (0.25 × 10^6^ cells) from female FVB-GFP mice (H2K^q^) were transplanted into HA mice (H2K^b^) through the tail vein within 30 h of acute liver injury, and the recipient mice were grouped as HAT-A. The T_reg_ cells from HA mice were primed against donor MHC antigens by co-culturing with splenic DCs of FVB mice for 48 h to generate allo-antigen sensitized T_reg_ cells (sT_reg_ cells). To establish transplant tolerance, allogeneic Lin^−^ BMCs and sT_reg_ cells were co-transplanted into HA mice, and the recipient mice were grouped as HAT-AT. **b** Plasma FVIII activity was measured by *in vitro* coagulation assay performed by using a Coatest assay kit. FVIII activity as percentage of normal plasma in wild-type mice is shown. There was no significant increase in FVIII activity in HAT-A mice but the activity was much higher in HAT-AT mice (*P* < 0.0001, HAT-A versus HAT-AT mice) after 3 and 6 months of transplantation. **c** Capillary blood clotting time. WT, HA, HAT-A, and HAT-AT mice were subjected to the assay after 3 months of transplantation. **d** Tail-clip challenge test. Survival of mice after 24 h of tail clip was determined, and HAT-AT group showed no mortality. *BM* bone marrow, *BMC* bone marrow cell, *DC* dendritic cell, *FVIII K/O* factor VIII knockout, *HA* hemophilia A, *HAT-A* hemophilia A mice transplanted with allogeneic cells, *HAT-AT* hemophilia A mice transplanted with allogeneic and regulatory T cells, *n* number of mice in each experiment, *sT*_*reg*_ sensitized regulatory T, *WT* wild-type

Transplantation of allogeneic Lin^−^ BMCs (FVB/J) and sT_reg_ cells was performed in HA mice as in the scheme in Fig. 1a after inducing acute centrilobular necrosis in the liver, as presented in Additional file 2: Figure S1. Phenotype correction of the recipient mice was assessed after 3 months of transplantation. The HA mice used in the study contain *neo* sequence in the exon 16 of factor VIII A3 domain, so intact FVIII (lc) protein is not expressed [17]. The plasma FVIII activity in HAT-AT and HAT-A mice after 3 and 6 months of transplantation was determined by Coatest assay. The activity in HAT-A mice was marginally improved to 2.9 ± 0.6 %, whereas a significant increase up to 16.1 ± 2.2 % of wild-type activity was obtained in HAT-AT mice (Fig. 1b). The plasma FVIII activity was retained even after 6 months of transplantation. The capillary blood clotting time in HAT-A mice was marginally decreased, whereas significant (*P* < 0.0001) reduction (2.9 ± 0.9 against 12.9 ± 0.9 min) was observed in the HAT-AT group as compared to HA mice (Fig. 1c). All 18 hemophilic mice that received allogeneic Lin^−^ BMCs with sT_reg_ cells (HAT-AT) achieved hemostasis after the tail-clip challenge. In the HAT-A group, most of the mice died because of excessive bleeding within 24 h of the tail-clip, as did HA mice (Fig. 1d). Phenotype correction in the HAT-AT group implies adequate FVIII production in HAT-AT mice to activate the intrinsic coagulation pathway when compared to HAT-A and HA mice.

### sT_reg_ cells exhibit enhanced regulatory effects than nT_reg_ cells on T cell proliferation *in vitro*

**Fig. 2:**
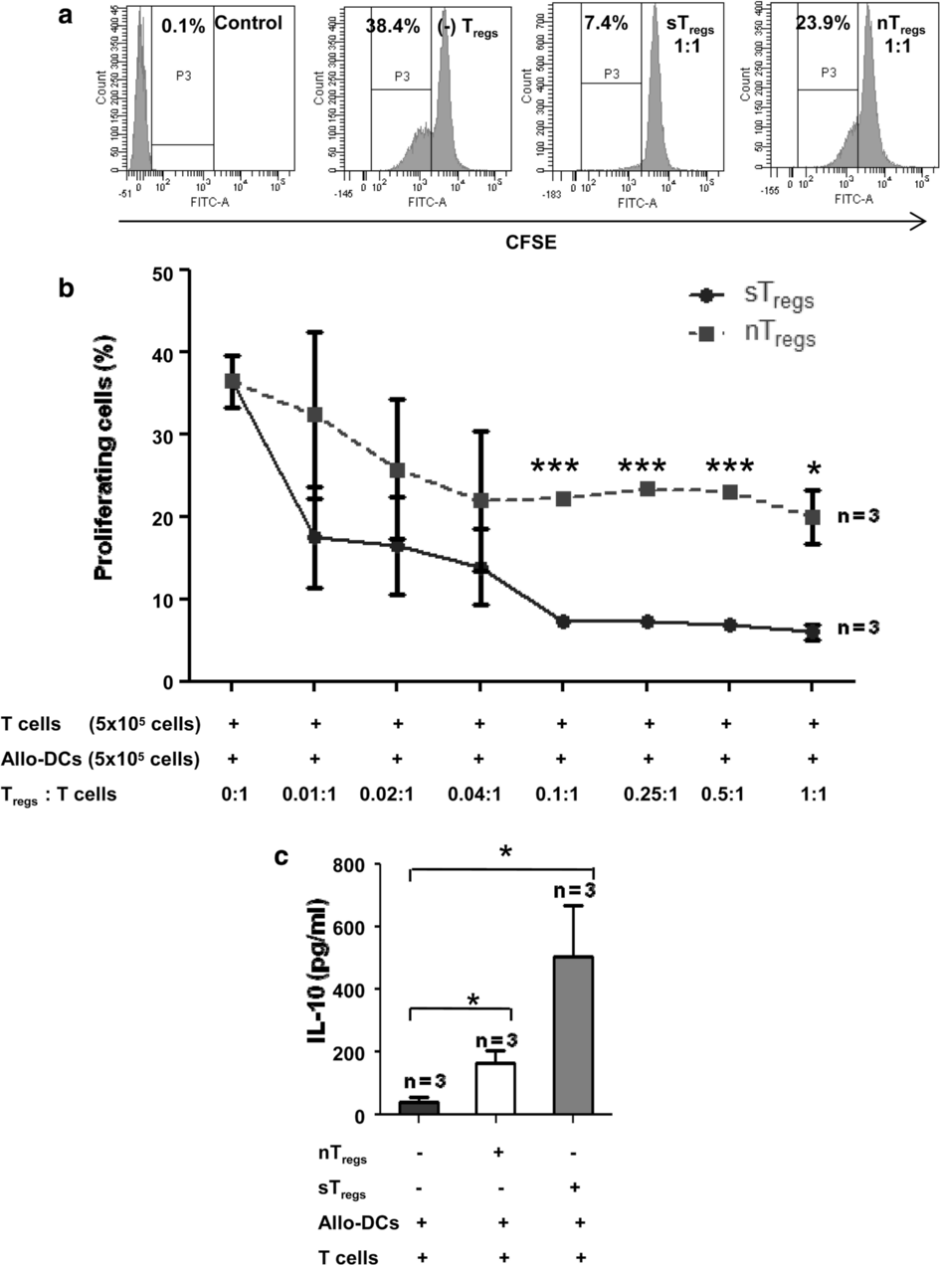
*In vitro* functional characterization of sT_reg_ cells. **a** T-cell suppression (CFSE dilution) assay. DCs (1 × 10^5^ cells) of FVB/J mice and 1 × 10^5^ CFSE-labeled CD4^+^CD25^−^ T cells (HA mice) were added to set up T-cell suppression assay. The nT_reg_ or sT_reg_ cells were added in each well at different ratios of T cells and cultured for 5 days. The proliferation of T cells in mixed lymphocyte reaction was measured in terms of dilution of CFSE intensity as shown in the representative histograms. **b** A significant (*P = 0.0142) difference in suppression of T-cell proliferation between two groups of T_reg_ cells was noticed at 0.1:1, 0.25:1, 0.5:1, and 1:1 ratios of the regulatory to effector T cells. **c** IL-10 secretion in the culture supernatants (T_reg_ cells: T cells = 1:1) was measured by enzyme-linked immunosorbent assay. A significant increase in IL-10 levels was observed in cultures with sT_reg_ cells (*P* = 0.04). *CFSE* carboxyfluorescein succinimidyl ester, *DC* dendritic cell, *HA* hemophilia A, *IL-10* interleukin-10, *n* number of experiments, *nT*_*reg*_ naïve regulatory T, *sT*_*reg*_ sensitized regulatory T, *T*_*reg*_ regulatory T (***) *P* < 0.0001, (*) *P* < 0.05

In the above experiments, sT_reg_ cells were used because of robust regulatory properties as compared to nT_reg_ cells. To know which of the two regulatory cell types was more potent, we conducted T-cell suppression assay. Splenic CD4^+^CD25^+^ T_reg_ cells of HA mice were sensitized to allo-DCs as before, and a proliferation assay was conducted by co-culturing CFSE-labeled T cells with different proportions of sensitized (sT_reg_) and naïve (nT_reg_) cells. The results showed a dose-dependent inhibition of T-cell proliferation by both regulatory cell types. The suppressive effect was pronounced (*P* < 0.001, 0.05) with sT_reg_ cells at ratios of 0.1:1 up to 1:1 to T cells when compared to nT_reg_ cells (Fig. 2a, b). There was no significant change in Foxp3 expression in the T_reg_ cells on antigen priming, and more than 90 % of the cells expressed the marker as in nT_reg_ cells, depicted in the dot-plots of Additional file 3: Figure S2.

As T_reg_ cells also suppress proliferation of immune reactive T cells by secretion of cytokines like IL-10 and transforming growth factor-beta, we determined IL-10 level in the culture supernatants of the above experiments. Interestingly, both nT_reg_ and sT_reg_ cells secreted IL-10, but the quantity was significantly higher in sT_reg_ cells as compared to nT_reg_ cells (Fig. 2c). Hence, the above results suggest that priming of T_reg_ cells with major histocompatibility complex (MHC) antigen of allogeneic DCs enhances their prospective regulatory effects on T cells during subsequent exposure of the same antigen.

### Allo-specific systemic immune modulation by sT_reg_ cells in HAT-AT mice

**Fig. 3:**
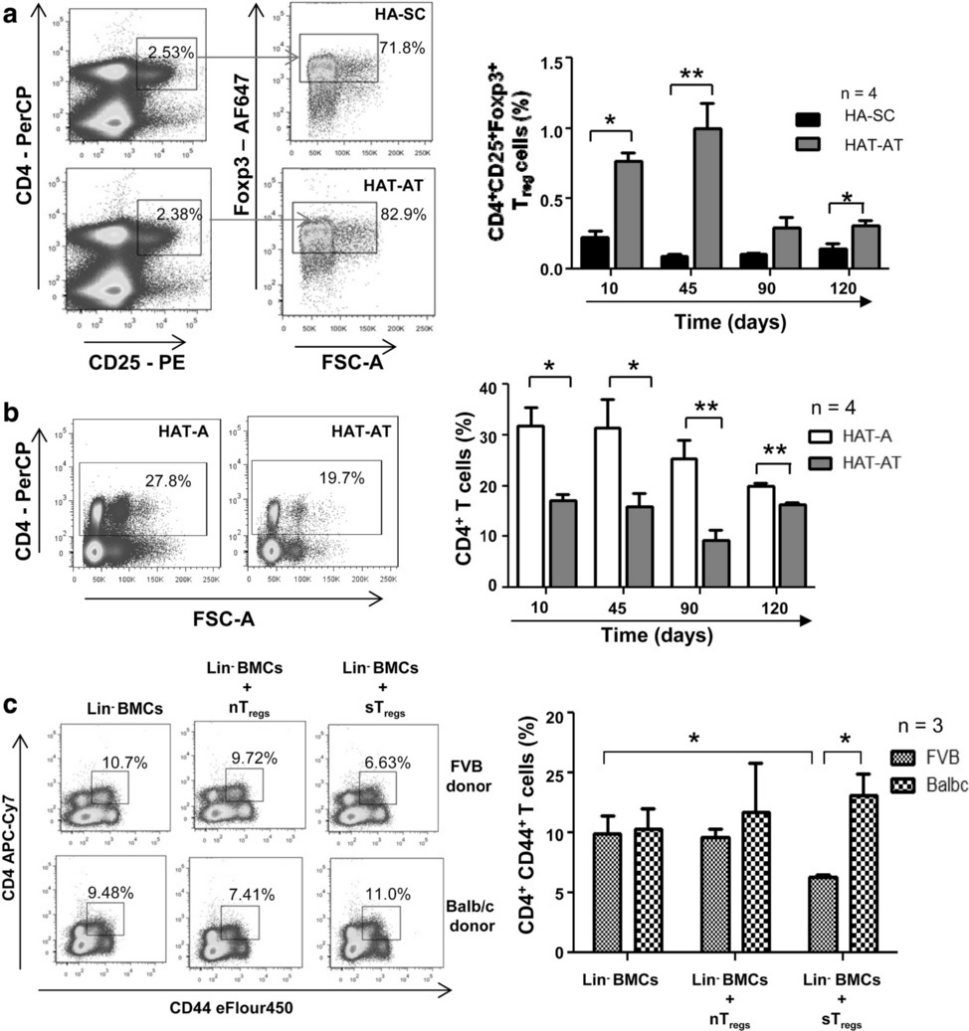
sT_reg_ cells modulate systemic immune response with allo-specificity *in vivo.* **a** Time course analysis of T_reg_ cells by flow cytometry in both HAT-AT and HA-SC mice. Representative dot-plots are shown in the *left panel*, and statistical analysis in the *right panel*. **b** CD4^+^ T cells in spleen of HAT-AT and HAT-A mice were analysed by flow cytometry. Representative dot-plots are shown in the *left panel*, and statistical analysis in the *right panel*. **c** Allo-specific activation of sT_reg_ cells. T-cell activation in response to allogeneic Lin^−^ BMCs of FVB-GFP and Balb/c mice were compared. Representative dot-plots show the levels of CD4^+^CD44^+^ T cells after 48 h of transplantation (*left panel*), and statistical analysis is shown in the *right panel* (*n* = 3). *P < 0.05, **P < 0.01. *BMC* bone marrow cell, *HA-SC* sham control mice in which liver was damaged but neither Lin^−^ bone marrow cells nor sensitized regulatory T cells were transplanted, *HAT-A* hemophilia A mice transplanted with allogeneic cells, *HAT-AT* hemophilia A mice transplanted with allogeneic and regulatory T cells, *n* number of mice in each experiment, *nT*_*reg*_ naïve regulatory T, *sT*_*reg*_ sensitized regulatory T, *T*_*reg*_ regulatory T

Time course analysis of CD4^+^CD25^+^Foxp3^+^ T_reg_ cells in the spleen of HAT-AT and HA-SC (sham control mice in which liver was damaged but neither Lin^−^ BMCs nor sT_reg_ cells were transplanted) mice showed a significant increase in the percentage of T_reg_ cells in the HAT-AT group in comparison to the HAT-SC group of mice within the first 2 months of transplantation, and the percentages later became comparable (Fig. 3a). Furthermore, the percentage of splenic CD4^+^ T cells in HAT-A mice was 31.9 ± 3.62 %, which significantly (*P* < 0.01) decreased to 17.13 ± 1.15 % in HAT-AT within 10 days of co-transplantation with sT_reg_ cells. Interestingly, this suppressive effect was continued throughout the course of the study (Fig. 3b). The above results suggest that effective immune suppression and development of tolerance are attributed to sT_reg_ cells.

To determine *in vivo* allo-specificity of sT_reg_ cells, an experiment was set up to compare CD4^+^ T-cell activation by allogeneic donor Lin^−^ BMCs from two different donor backgrounds (Balb/c and FVB/J) in the presence of FVB-specific sT_reg_ cells. After 2 days of transplantation, the levels of antigen-experienced CD4^+^CD44^+^ activated T cells in the spleen were analysed, and the basal level in the spleen of normal mice was 0.95 ± 0.07 % (data not shown). We observed a significant (*P* < 0.05) reduction of activated CD4^+^CD44^+^ T cells in the case of HAT-AT (FVB/J donor) as compared to HAT-A (FVB/J donor). In the parallel experiments, the suppression of T-cell activation against Lin^−^ BMCs from Balb/c mice (third-party donor) was insignificant (*P* = 0.44) in the presence of FVB-specific sT_reg_ cells. There was a significant reduction in the levels of CD4^+^CD44^+^ T cells in HAT-AT mice (FVB/J donor) when compared with HAT-AT mice (Balb/c donor) in the presence of FVB-specific sT_reg_ cells (Fig. 3c). These results indicate that sT_reg_ cells exhibit allo-specificity *in vivo*.

### sT_reg_ cells suppress immune reactions in recipient liver

**Fig. 4:**
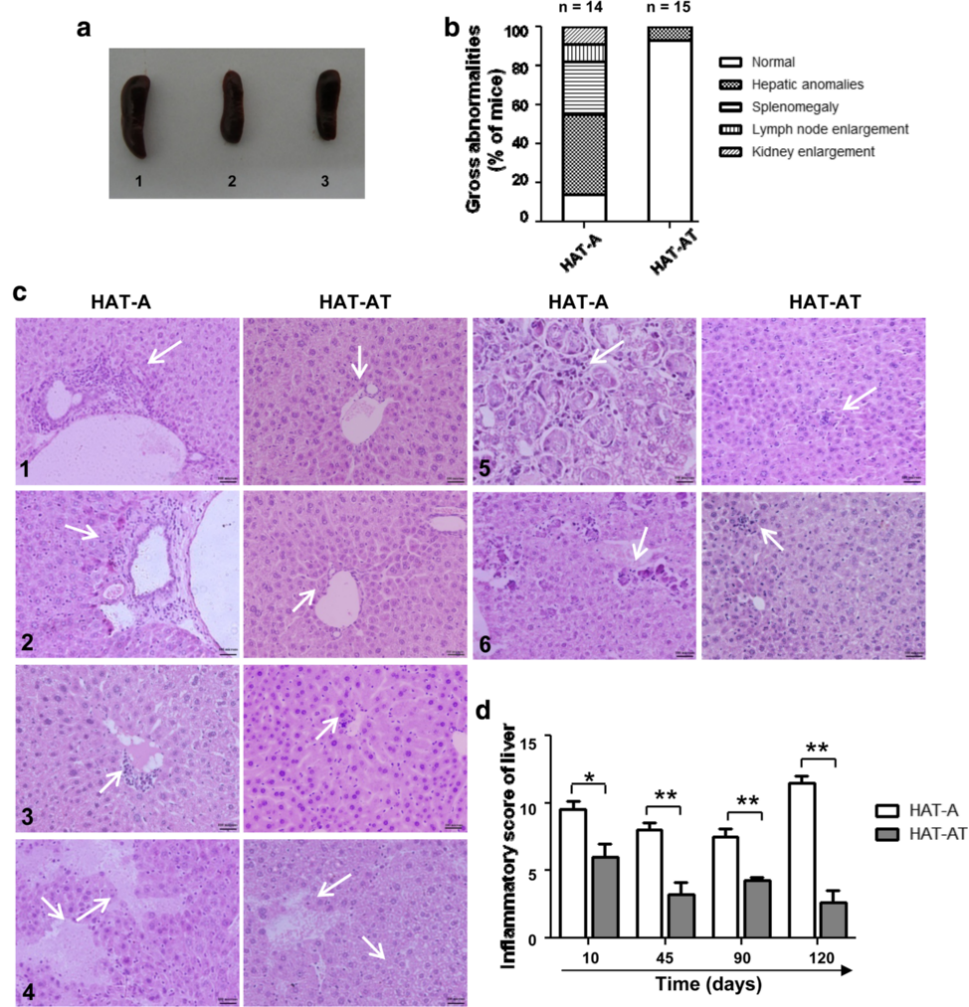
Sensitized regulatory T cells induce suppression of inflammatory responses in the liver. **a** Splenomagaly in HAT-A (1) mice in comparison with HAT-AT (2) and HA (3) mice. **b** Gross macroscopic abnormalities in mice. Mice with anomalies in various organs were counted, and percentage of mice with each anomaly was calculated. Most of the HAT-A mice showed abnormalities with respect to two to three indicators, whereas more than 90 % of the HAT-AT mice were normal. **c** Inflammation in the liver by histopathological analysis of HAT-A and HAT-AT mice after 120 days of transplantation. Representative images are shown for scoring of various inflammatory parameters: (1) portal inflammation, (2) periportal necro-inflammation, (3) endotheliasis, (4) necrosis, (5) sinusoidal lymphocyte infiltration, and (6) lobular necro-inflammation. *Arrow* indicated different immunoreactions with respect to the said parameters (scale of 100 μm; 200× magnification). **d** Comparative inflammatory scoring on the basis of histopathological analysis of the liver at different time intervals after transplantation in HAT-A and HAT-AT groups (*P < 0.05, **P < 0.01). *HAT-A* hemophilia A mice transplanted with allogeneic cells, *HAT-AT* hemophilia A mice transplanted with allogeneic and regulatory T cells, *n* number of mice in each time point

Examination of gross abnormalities in the liver, spleen, kidney, and lymph nodes of HAT-A and HAT-AT mice showed that inflammatory responses were largely exhibited only in the HAT-A group (Fig. 4a, b). The immune response against allograft can lead to infiltration of T cells into the graft site (in this case, the liver) and this may lead to persistent inflammatory reactions [18] that can result in cellular rejection. The extent of inflammatory response in the liver was ascertained by scoring various parameters [16] observed in HAT-A and HAT-AT mice (Fig. 4c and histopathological scorings mentioned in Additional file 1: Table S1) at various time intervals after transplantation. Among these, bile duct inflammation was rarely observed in our study. Histopathological scoring showed a significant decline of inflammatory reactions in the HAT-AT group when compared to the HAT-A group (Fig. 4d). These preliminary results indicate that sT_reg_ cells can circumvent inflammatory responses in the target site of engraftment, such as the liver in this case.

**Fig. 5:**
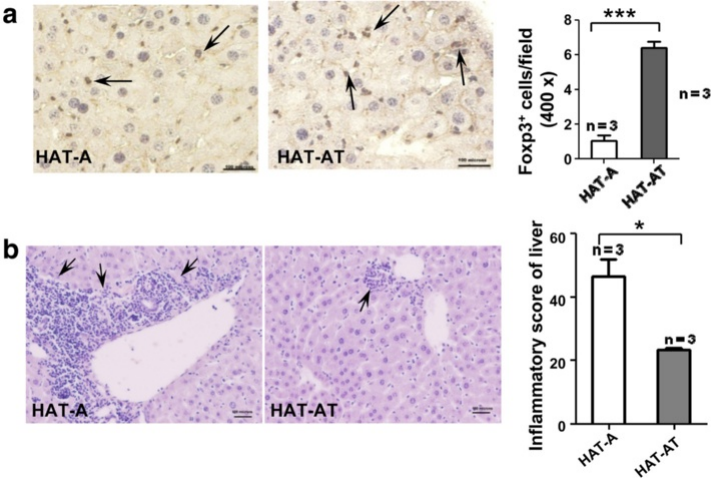
Long-term retention of transplant tolerance. **a** Migration of regulatory T cells to the liver. Representative photomicrographs show Foxp3-expressing cells (*left panel*, *arrows*) after 3 months of transplantation (scale of 100 μm; 400× magnification). Statistical analysis is shown in the *right panel*. **b** After 1 year of primary transplantation, a second dose of allogeneic 0.25 × 10^6^ Lin^−^ BMCs from FVB-GFP cells was given to HAT-A and HAT-AT mice after liver injury was inflicted. Representative photomicrographs show extensive immune reactions in HAT-A mice (scale of 100 μm; 200× magnification). Statistical analysis of inflammatory scores determined on the basis of histo-pathological analysis of liver is shown in the *right panel. BMC* bone marrow cell, *HAT-A* hemophilia A mice transplanted with allogeneic cells, *HAT-AT* hemophilia A mice transplanted with allogeneic and regulatory T cells, *n* number of mice in each experiment (***) *P* < 0.0001, (*) *P* < 0.05

As migration of T_reg_ cells to the site of engraftment can confer protection to allograft against cytotoxic effects of infiltrating T cells, we examined their presence in different liver lobes. Morphometric analyses confirmed the presence of an average of six Foxp3^+^ cells per field in HAT-AT mice liver sections and this was significantly (*P* < 0.0001) higher than that in HAT-A mice (Fig. 5a). To determine the long-term immune suppression by sT_reg_ cells, after 1 year of transplantation, HAT-A and HAT-AT mice were given a second dose of 0.25 × 10^6^ Lin^−^ BMCs (FVB/J donor) after liver injury was induced. An intense inflammatory response was evidenced in the liver of HAT-A mice as compared to HAT-AT mice as shown in Fig. 5b (*P* = 0.05). The inflammatory score in HAT-AT mice after secondary challenge with allogeneic cells (Fig. 5b) was much higher than that observed in the earlier experiment (Fig. 4d) because of aging of mice and inflicting second time injury. These results indicate that immune suppression in the liver by sT_reg_ cells can reduce the cellular responses involved in graft rejection even over a period of 1 year.

### Donor-derived endothelial cells are the major contributor of FVIII in HAT-AT mice

**Fig. 6:**
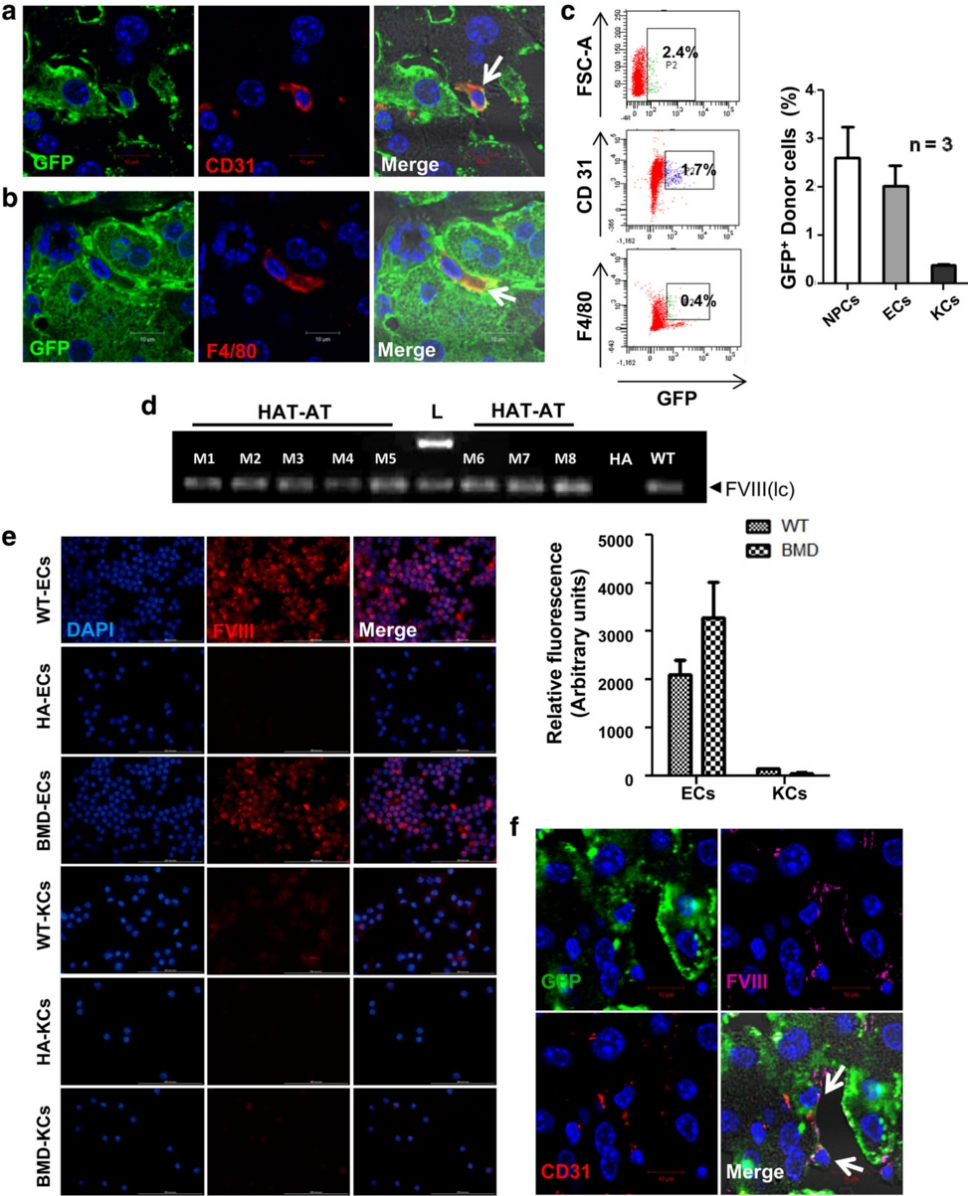
FVIII synthesis by donor-derived endothelial cells and Kupffer cells in HAT-AT liver. **a** Donor-derived endothelial cells indicated by white arrow (anti-GFP/donkey anti-mouse Alexa fluor 488 and anti-CD31/donkey anti-rat Alexa fluor 594) in HAT-AT liver (scale of 10 μm; 630 × 2.8 zoomed magnification). **b** Donor-derived Kupffer cells indicated by white arrow (anti-GFP/donkey anti-mouse Alexa fluor 488 and anti-F4/80/donkey anti-rat Alexa fluor 594) in HAT-AT liver (scale of 10 μm; 630 × 2.8 zoomed magnification). **c** Flow cytometric analysis of donor cells in non-parenchymal fraction of HAT-AT mouse liver after 1 month of transplantation. Representative dot-plots are given in the *left panel*, and quantitative analysis is shown in the *right panel* (*n* = 3). **d** FVIII(lc) gene expression in livers of HAT-AT, HA, and WT mice. **e** Immunocytochemical analysis of FVIII in endothelial and Kupffer cells from HA, WT, and HAT-AT mice (scale of 100 μm; 600× magnification). Relative cell fluorescence intensities are shown in the bar diagram on *right*. **f** Highly magnified confocal images (scale of 10 μm; 630× 2.8 zoomed magnification) show that donor-derived endothelial cells express FVIII indicated by white arrow (anti-GFP/donkey anti-mouse Alexafluor 488, anti-CD31/donkey anti-rat Alexafluor 594, anti-FVIII(lc)/donkey anti-rabbit Alexafluor 647). *BMD* bone marrow derived, *DAPI* 4′,6-diamidino-2-phenylindole, *EC* endothelial cell, *GFP* green fluorescent protein, *FVIII* factor VIII, *FVIII(lc)* factor VIII light chain, *HA* hemophilia A, *HAT-AT* hemophilia A mice transplanted with allogeneic and regulatory T cells, *KC* Kupffer cell, *n* number of mice in each experiment, *NPC* non-parenchymal cell, *WT* wild-type

Among the non-parenchymal cells, Lin^−^ BMCs were found to generate mainly CD31-expressing ECs (Fig. 6a). A few F4/80-expressing Kupffer cells (KCs) were also detected in the recipient liver (Fig. 6b). Earlier, it has been shown that in liver FVIII is synthesized primarily by LSECs but that a small amount can be synthesized by KCs. The contribution of donor cells to LSEC and KC fractions in HAT-AT mice liver was ascertained by flow cytometric analysis. It was revealed that GFP^+^ donor cells constituted 2.6 ± 0.64 % of total non-parenchymal fraction, of which 2.02 ± 0.42 % was CD31^+^ ECs and 0.37 ± 0.02 % was F4/80^+^ macrophages/KCs (Fig. 6c). The time course analysis indicated that there was an increase of GFP^+^CD31^+^ ECs in the non-parenchymal fraction of the liver after initial engraftment; this is presented in the dot-plot analysis in Additional file 4: Figure S3. Further analysis of Ki67 antigen revealed that these donor-derived ECs also underwent proliferation along with host ECs. The percentages of proliferation of host and donor-derived ECs are shown in Additional file 5: Figure S4.

After 3 months of transplantation, FVIII expression in the liver of HAT-AT mice was analysed. The HA mice used in the study had a *neo* sequence in the exon 16 of factor VIII A3 domain preventing the synthesis of FVIII light chain (lc) protein and so primers were specifically designed against this region. Thus, amplification did not occur in HA mice samples, whereas FVIII(lc) expression was observed in wild-type and HAT-AT liver samples (Fig. 6d).

To decipher the role of donor-derived cells in correction of HA, we first analysed relative FVIII transcript levels in few specific liver non-parenchyma as well as BMC fractions of wild-type mice with respect to donor Lin^−^ BMCs. Among all of the cell types analysed, LSECs apparently expressed the highest level of FVIII(lc) transcript, followed by KCs, CD45^−^ (stromal cells), and CD11b^+^ BMCs; relative fold changes of gene expression are depicted in Additional file 6: Figure S5. Next, BM-derived cells (GFP^+^CD31^+^ and GFP^+^F4/80^+^) were isolated from the liver of HAT-AT mice and immunocytochemically analysed for the expression of FVIII(lc) protein. As shown in Fig. 6e, LSECs isolated from HA mice did not express FVIII(lc) protein, whereas wild-type LSECs and ECs isolated from HAT-AT mice express the same. A similar trend was observed in wild-type KCs and eGFP^+^F4/80^+^ cells, isolated from HAT-AT mice liver, but the expression level was much lower. To compare FVIII(lc) protein expression in liver resident LSECs and KCs (in wild-type mice) with BM-derived ECs and KCs (in HAT-AT mice), we have determined relative fluorescence intensity of the protein by ImageJ software. It was apparent that FVIII(lc) protein level was significantly high in both LSECs and BM-derived ECs but much lower in KCs (Fig. 6e, bar diagram). On the basis of above results, we propose that BM-endothelial progenitor cells were converted into FVIII-expressing LSEC-like cells in the recipient mice liver. To prove that LSECs and BM-derived ECs were functionally similar, we conducted an LDL uptake study; the results revealed that LDL uptake was comparable, as presented in Additional file 7: Figure S6. To confirm that engrafted BM-derived ECs in liver synthesized FVIII, we conducted immunohistochemistry analysis in HAT-AT liver sections after 4 months of transplantation. The results clearly demonstrate that GFP^+^CD31^+^ cells expressed FVIII (Fig. 6f). These results suggest that transplantation of Lin^−^ BMCs causes phenotype correction in HA mice because of the synthesis of FVIII by BM-derived ECs.

### sT_reg_ cells support allogeneic BMC-mediated liver regeneration and functional improvement

**Fig. 7:**
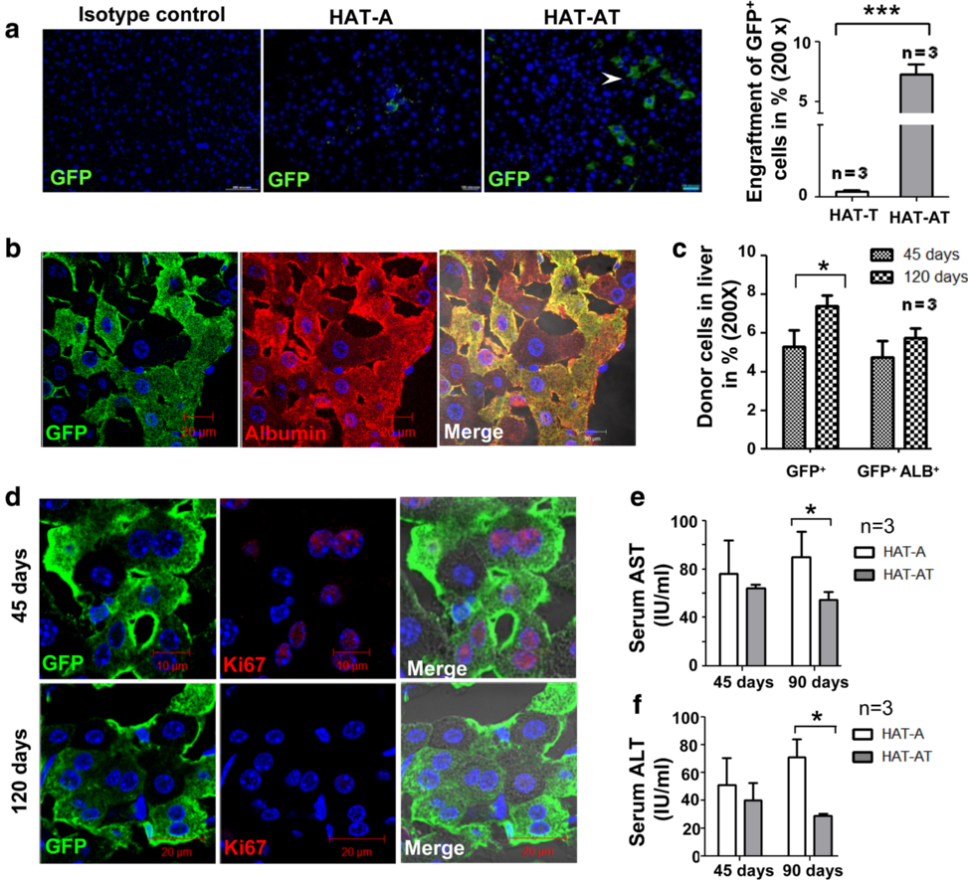
Engraftment and regeneration of liver by donor-derived hepatocytes. **a** Representative photomicrographs show immunohistochemistry analysis of liver sections of HAT-A and HAT-AT mice after 4 months of transplantation. Sections were stained with anti-GFP/donkey anti-mouse Alexafluor 488 (scale of 100 μm; 200× magnification). Statistical analysis of GFP^+^ cells (indicated by *white arrow*) is shown in the *right panel*. **b** Immunohistochemical analysis of HAT-AT mouse liver sections after 4 months of transplantation. Representative images (scale of 20 μm; 630× magnification) show donor derived hepatocytes (anti-GFP/donkey anti-mouse Alexa fluor 488 and anti-albumin/donkey anti-goat Alexa fluor 594). **c** Quantitative analysis of GFP^+^ and GFP^+^albumin^+^ cells in HAT-AT liver (200× magnification) after 45 and 120 days of transplantation (*P = 0.03). **d** Representative images for Ki67^+^ donor cells in HAT-AT liver after 45 and 120 days of transplantation (scale of 10, 20 μm; 630 × 2.8 magnification). **e** Analysis of serum AST levels in HAT-AT and HAT-A mice after 45 and 90 days of transplantation. **f** Analysis of serum ALT levels in HAT-AT and HAT-A mice after 45 and 90 days of transplantation. *ALB* albumin, *ALT* alanine aminotransferase, *AST* aspartate aminotransferase, *GFP* green fluorescent protein, *HAT-A* hemophilia A mice transplanted with allogeneic cells, *HAT-AT* hemophilia A mice transplanted with allogeneic and regulatory T cells, *IU* international units, *n* number of mice in each experiment (***) *P* < 0.0001, (*) *P* < 0.05

Transplant tolerance generated by sT_reg_ cells can improve engraftment of donor cells in the liver. So liver sections of HAT-A and HAT-AT mice were analysed for engraftment of eGFP-expressing donor cells after 4 months of transplantation. Immunohistochemical analysis of recipient liver sections showed that, owing to suppression of immune response, engraftment of more allogeneic BMCs occurred in HAT-AT mice liver as compared to HAT-A (Fig. 7a). Morphometric analysis based on 30 non-overlapping fields in each set of mice showed that the percentage of GFP^+^ cells in HAT-AT liver was 7.27 ± 0.82 %, which is significantly higher (*P* < 0.0001) than that in HAT-A liver (Fig. 7a, bar diagram). On further analysis, it was found that many donor-derived cells expressed albumin and attained morphology similar to that of hepatocytes (Fig. 7b). Morphometric analyses of images (200× magnification) in 20 different fields of liver sections per mouse after 45 and 120 days of transplantation showed that 5.26 ± 0.88 % and 7.57 ± 0.47 % cells, respectively, were eGFP-expressing. The percentages of donor-derived albumin-expressing cells in these two time points were 4.72 ± 0.87 % and 5.72 ± 0.5 %, respectively (Fig. 7c, bar diagram).

Since donor-derived cells increased in their number, we were interested to know how long they can multiply. So liver sections were co-stained for eGFP and Ki67 antigens after 45 and 120 days of transplantation. It was revealed that some donor-derived cells that morphologically resembled hepatocytes underwent proliferation in the 45th day samples, but no Ki67 staining was observed in the liver after 120 days of transplantation (Fig. 7d). As the donor-derived hepatocytes stopped proliferation in a later time point, we concluded that liver regeneration was completed.

Acute liver injury due to administration of toxic doses of acetaminophen results in increased serum alanine aminotransferase (ALT) and aspartate aminotransferase (AST) levels. These serum parameters were gradually normalized during liver regeneration. Owing to persistent inflammatory reactions for chronic rejection, the serum AST and ALT levels were maintained significantly high in HAT-A mice even after 90 days of transplantation. As expected, they decreased to normal levels in HAT-AT mice by this period (Fig. 7e, f).

## Discussion

In developed countries, factor replacement therapy is readily available, but owing to high prophylactic treatment cost, many patients are still treated on demand. The patients in the developing countries, owing to extreme financial constraint, are treated mostly on demand [19]. So a permanent solution for HA has been sought for quite some time. Gene therapy appears to be promising as a small increase in plasma factor levels can essentially improve the clinical outcome of HA [20]. Clinical studies have shown that 10–40 % of patients with the severe form of HA develop inhibitors and thus treatment fails in the long run [21]. In humans, the primary source of plasma FVIII is LSECs [1–3]; however, the same has been found to be synthesized in low quantities by mesenchymal stem cells (MSCs) [22, 23] and macrophages [23].

Various cell-based therapies have been advocated for the treatment of HA in experimental animals, and in all cases therapeutic correction levels of plasma FVIII were achieved. Previously, we showed that syngeneic BM-derived uncommitted cells can synthesize FVIII and confer protection against bleeding to death [12, 24]. Here, we have identified three different cell types—ECs, KCs, and hepatocytes—that can originate from allogeneic Lin^−^ BM-derived cells in recipient mouse liver. Interestingly, we observed that FVIII synthesis occurred primarily in BM-derived ECs. Owing to the shortage of cadaver livers, therapeutic correction of HA by transferring healthy LSECs [8] may not be feasible. Lin^−^ BMCs contain endothelial progenitors [25, 26]; so we propose that these can ultimately differentiate into functional LSECs [27] and produce FVIII. Lin^−^ BMCs also consist of MSCs, which can participate directly in liver regeneration by secreting factors that help in tissue repair, modulation of inflammatory responses, and activation of hepatic progenitor cells [28]. An earlier report suggested that BM-MSCs and mononuclear cells in peripheral blood secrete FVIII and can act as a supporting system to deliver FVIII at the bleeding sites [23]. Even though MSCs can express FVIII protein [22], they have been used for short-term transplantation study of phenotype conversion in HA mice [23].

Our study confirmed the majority of BM-derived cells as hepatocyte-like cells, as a substantial fraction of necrosed hepatocytes were replaced by them [12]. Even though BM-derived hepatocytes have been reported in liver regeneration [12, 29, 30], the plasticity of BM stem cells had been questioned by many groups. The general belief is that cell fusion is the principal mechanism for generation of BM-derived hepatocytes [31–34]. Even if BMCs assume hepatic phenotype by fusion followed by ploidy reduction or by direct differentiation, our approach still has a high clinical relevance in the context of liver regeneration using allogeneic BMCs. In acetaminophen-induced liver injury, we observed that donor cells were engrafted mostly in the liver rather than in the BM or spleen (~0.1 %). In contrast, in the case of a radiation-induced injury model, substantial fractions of BMCs are found to be engrafted in the BM and spleen because of their natural niches. Thus, we conclude that Lin^−^ BMCs may act as an ideal source of cells for liver regeneration in HA mice.

A highly competent core system involving FVIII-expressing LSECs is more desirable so that repeated infusion of purified FVIII will not be required. Moreover, since liver is an immune-privileged organ, the engrafted Lin^−^ BMCs can facilitate long-term management of the disease. In the present model, transplantation of Lin^−^ BMCs into HA mice restored plasma FVIII activity well above the therapeutic correction level (≤10 % normal activity). Another unique advantage was that no FVIII inhibitor was detected in our earlier study using the same cells but from a syngeneic source [12]. Anti-FVIII antibody (inhibitor) formation in the long run occurs both in cases of recombinant and plasma-derived FVIII therapy and in the experimental FVIII gene therapy model [7], which urged for alternate immunological approaches to prevent these humoral responses [35–37]. We propose that in perturbed liver a fraction of the damaged hepatocytes, LSECs, and KCs are replaced by BM-derived cells. High expression of FVIII by BM-derived ECs suggests that endothelial progenitor cells of the BM might have differentiated into liver-specific endothelial-like cells. We observed relatively high expression of FVIII by these cells as compared with wild-type LSECs and this may be due to high selection pressure in HA mice for the synthesis of FVIII. This could explain why plasma FVIII activity in HAT-AT mice was not commensurate with the number of BM-derived ECs engrafted in the liver.

As hemophilia is a genetic disease, autologous cells cannot be used for treatment unless they are genetically modified to express the desired protein. After allogeneic transplantation, the host T lymphocytes upon activation with donor antigens acquire effector functions and destroy the donor cells as was evidenced by low retention of donor cells in HAT-A mice. Allo-graft rejection is mediated by three effector pathways: (a) delayed-type hypersensitivity (DTH) reactions by allo-antigen activated CD4^+^ T cells, (b) activation of CD8^+^ cytotoxic T lymphocytes, and (c) antibody-mediated. Since the liver is the major site for engraftment of the allogeneic Lin^−^ BMCs, inflammatory reactions were pronounced in the liver. The T-cell response was evidenced by low retention of donor cells in HAT-A mice. In this study, we examined the response of CD4^+^ T cells to MHC antigens on allogeneic donor cells and their suppression by establishing peripheral tolerance using T_reg_ cells. We have demonstrated that sT_reg_ cells can suppress immune responses against allogeneic BMCs by reducing inflammatory response in the liver, thus leading to better engraftment of cells. Our finding concurred with other reports in which allo-antigen-specific T_reg_ cells improved peripheral tolerance to BM, skin, and cardiac allo-grafts [38, 39].

During sensitization, T_reg_ cells were primed against MHC molecules of the donor cells. In the host, these sT_reg_ cells, through linked suppression, not only recognize a single allo-antigen exposed during *in vitro* sensitization but also can suppress immune responses against most allo-antigens expressed by the graft [40]. These activated T_reg_ cells in circulation can also induce secondary regulatory phenotype in circulatory T cells by infectious tolerance and generate induced T_reg_ cells, which can further lead to effective immune suppression during the rejection phase [41]. Furthermore, we conclude that contact-independent mechanisms were also responsible for controlling immune response as IL-10 was detected in T-cell proliferation assay [42, 43]. Another benefit of adoptive transfer of *in vitro* antigen primed T_reg_ cells was that their suppressive effects were comparatively antigen-specific as no significant activation of CD4^+^ T cells was observed when sT_reg_ cells were co-transplanted with third-party donor Lin^−^ BMCs. Not only that, our preliminary results suggested retention of peripheral tolerance for long duration with a single dose of sT_reg_ cells. The presence of significantly higher numbers of T_reg_ cells in recipient liver proved that either sT_reg_ cells or induced T_reg_ cells, derived from T-cell pool, or both can migrate to the primary site of inflammation to prevent rejection of allogeneic cells. Long-term management of HA by the synthesis of prophylactic levels of FVIII by donor-derived cells can overcome the requirement for repeated infusion of FVIII in clinical settings. Furthermore, this approach will allow us to overcome biosafety issues pertaining to gene therapy. In future adoptive transfer of donor MHC, primed T_reg_ cells may substitute for the use of immunosuppressive drugs in case of transplantation of allogeneic cells. If the present approach has to be experimented in higher animal models of HA like Chapel Hill dogs, a pre-conditioning regimen of FVIII is recommended to protect the recipient against initial hemorrhage while inducing partial liver injury. Our preliminary results suggests that T_reg_ cells can be taken from the recipient and primed against donor antigens and expanded *in vitro* to obtain enough cells for exerting immune suppressive effects. Besides establishing a pre-conditioning FVIII regimen, further studies need to be pursued on long-term retention of allo-specificity of these primed T_reg_ cells before bench-to-bedside translation.

## Conclusions

Our report establishes that transfer of allo-antigen primed T_reg_ cells along with allogeneic Lin^−^ BMCs in a liver regeneration model of HA mice allows engraftment of BM-derived endothelial progenitor cells in liver, synthesis of FVIII by donor-derived cells, and subsequent correction of bleeding phenotype. Engraftment of allogeneic cells in the liver was possible because of systemic immune suppression and reduction in inflammatory reactions in the liver, the latter of which promotes generation of allo-antigen-specific transplantation tolerance. Furthermore, donor BM-derived hepatocytes play an important role in regeneration of injured liver parenchyma.

## Additional files

Additional file 1: Table S1. Histopathological scoring of inflammatory reactions in liver of transplanted mice. *HAT-A* hemophilia A mice transplanted with allogeneic cells, *HAT-AT* hemophilia A mice transplanted with allogeneic and regulatory T cells. (DOC 60 kb) Additional file 2: Figure S1. Histopathology of liver sections after 24 h of acetaminophen-induced injury. (TIFF 1189 kb) Additional file 3: Figure S2. Expression of Foxp3 in naïve (nT_reg_) and allo-sensitized (sT_reg_) regulatory T cells. (TIFF 523 kb) Additional file 4: Figure S3. Donor-derived non-parenchymal cells in HAT-AT liver. **a** Time course analysis of GFP^+^ donor cells in non-parenchymal cells of HAT-AT mice. **b** Time course analysis of GFP^+^ donor-derived endothelial cells in non-parenchymal cells of HAT-AT mice. *GFP* green fluorescence protein, *HAT-AT* hemophilia A mice transplanted with allogeneic and regulatory T cells. (TIFF 643 kb) Additional file 5: Figure S4. Proliferation of endothelial cells in HAT-AT liver after 5 and 10 days of transplantation. Proliferating cells (Ki67^**+**^) were found to be present in both host and donor-derived fractions of endothelial cells. *HAT-AT* hemophilia A mice transplanted with allogeneic and regulatory T cells. (TIFF 733 kb) Additional file 6: Figure S5. Expression of FVIII mRNA in liver and bone marrow cells of wild-type mice. Fold change calculated with respect to expression in Lin^−^ BMCs. *BMC* bone marrow cell, *FVIII* factor VIII, *LSEC* liver sinusoidal endothelial cell. (*) *P* < 0.05. (TIFF 183 kb) Additional file 7: Figure S6. DiI-Ac-LDL (*) *P* < 0.05 uptake in WT LSECs and donor BMD ECs from HAT-AT liver. *BMD-EC* bone marrow-derived endothelial cell, *DiI-Ac-LDL* 1,19-dioctadecyl-3,3,39,39-tetramethylindo-carbocyanine perchlorate, *HAT-AT* hemophilia A mice transplanted with allogeneic and regulatory T cells, *WT LSEC* wild-type liver sinusoidal endothelial cell. (TIFF 491 kb)

## Abbreviations

ALT: Alanine aminotransferase
AST: Aspartate aminotransferase
BM: Bone marrow
BMC: Bone marrow cell
BSA: Bovine serum albumin
CFSE: Carboxyfluorescein succinimidyl ester
DC: Dendritic cell
EC: Endothelial cell
eGFP: Enhanced green fluorescent protein
FVIII: Factor VIII
FVIII(lc): Factor VIII light chain
HA: Hemophilia A
HAT-A: Hemophilia A mice transplanted with allogeneic cells
HAT-AT: Hemophilia A mice transplanted with allogeneic and regulatory T cells
IL-10: Interleukin-10
KC: Kupffer cell
LDL: Low-density lipoprotein
LSEC: Liver sinusoidal endothelial cell
MHC: Major histocompatibility complex
MSC: Mesenchymal stem cell
nT_reg_: Naïve regulatory T
PBS: Phosphate-buffered saline
PCR: Polymerase chain reaction
sT_reg_: Sensitized regulatory T
T_reg_: Regulatory T
