# Supplementary material for: Donor antigen-primed regulatory T cells permit liver regeneration and phenotype correction in hemophilia A mouse by allogeneic bone marrow stem cells
Source: Stem Cell Res Ther. 2015 Jul 8;6(1):129. doi: 10.1186/s13287-015-0119-9 (PMC4513683; doi:10.1186/s13287-015-0119-9)
Supplement: Additional file 1: Table S1. — Histopathological scoring of inflammatory reactions in liver of transplanted mice. HAT-A hemophilia A mice transplanted with allogeneic cells, HAT-AT hemophilia A mice transplanted with allogeneic and regulatory T cells. (DOC 60 kb) [file 13287_2015_119_MOESM1_ESM.doc]

**Supplementary Table 1: Histo-pathological scoring of inflammatory reactions in liver of transplanted mice**

| **Sl. No.** | **Parameters analysed** | **10 days** | | | | | | | **45 days** | | | | | | | | **90 days** | | | | | | | | **120 days** | | | | | |
| --- | --- | --- | --- | --- | --- | --- | --- | --- | --- | --- | --- | --- | --- | --- | --- | --- | --- | --- | --- | --- | --- | --- | --- | --- | --- | --- | --- | --- | --- | --- |
|  |  | **HAT-A** | | | | **HAT-AT** | | | **HAT-A** | | | | **HAT-AT** | | | | **HAT-A** | | | | **HAT-AT** | | | | **HAT-A** | | | **HAT-AT** | | |
|  |  | M1 | M2 | M3 | M4 | M1 | M2 | M3 | M1 | M2 | M3 | M4 | M1 | M2 | M3 | M4 | M1 | M2 | M3 | M4 | M1 | M2 | M3 | M4 | M1 | M2 | M3 | M1 | M2 | M3 |
| 1 | Portal Inflammation | 2 | 1 | 1 | 1 | 1 | 1 | 1 | 1 | 0 | 1 | 1 | 1 | 1 | 0 | 1 | 1 | 1 | 1 | 1 | 1 | 1 | 1 | 1 | 2 | 2 | 2 | 1 | 1 | 0 |
| 2 | Bile duct inflammation | 1 | 2 | 0 | 1 | 0 | 0 | 1 | 0 | 0 | 1 | 2 | 0 | 0 | 0 | 0 | 1 | 1 | 1 | 1 | 0 | 0 | 0 | 0 | 2 | 2 | 1 | 0 | 0 | 0 |
| 3 | Periportal necroinflammatory activity | 2 | 1 | 1 | 1 | 1 | 1 | 1 | 1 | 0 | 0 | 0 | 0 | 0 | 0 | 0 | 2 | 2 | 2 | 2 | 0 | 0 | 0 | 1 | 1 | 2 | 2 | 0 | 0 | 0 |
| 4 | Lobular necroinflammatory activity | 1 | 1 | 0 | 0 | 0 | 1 | 1 | 1 | 3 | 1 | 1 | 0 | 0 | 0 | 0 | 0 | 1 | 0 | 1 | 0 | 0 | 0 | 0 | 1 | 1 | 1 | 0 | 0 | 0 |
| 5 | Necrosis | 2 | 2 | 2 | 2 | 0 | 1 | 0 | 1 | 2 | 2 | 1 | 0 | 0 | 1 | 1 | 1 | 1 | 1 | 1 | 1 | 1 | 2 | 2 | 0 | 0 | 2 | 1 | 0 | 1 |
| 6 | Endotheliasis | 1 | 1 | 2 | 2 | 0 | 1 | 1 | 1 | 0 | 2 | 2 | 0 | 1 | 0 | 1 | 1 | 1 | 1 | 1 | 1 | 1 | 1 | 1 | 2 | 2 | 1 | 1 | 1 | 0 |
| 7 | Sinusoidal lymphocyte infiltration | 2 | 2 | 2 | 2 | 2 | 2 | 2 | 2 | 2 | 2 | 2 | 0 | 2 | 2 | 2 | 2 | 2 | 2 | 2 | 1 | 1 | 2 | 2 | 3 | 3 | 2 | 1 | 1 | 0 |
|  | **Total Score** | **11** | **10** | **8** | **9** | **4** | **7** | **7** | **7** | **7** | **9** | **9** | **1** | **4** | **3** | **5** | **6** | **7** | **8** | **9** | **4** | **4** | **4** | **5** | **11** | **12** | **11** | **4** | **3** | **1** |
