# Supplementary figures and images for: Donor antigen-primed regulatory T cells permit liver regeneration and phenotype correction in hemophilia A mouse by allogeneic bone marrow stem cells
Source: Stem Cell Res Ther. 2015 Jul 8;6(1):129. doi: 10.1186/s13287-015-0119-9 (PMC4513683; doi:10.1186/s13287-015-0119-9)

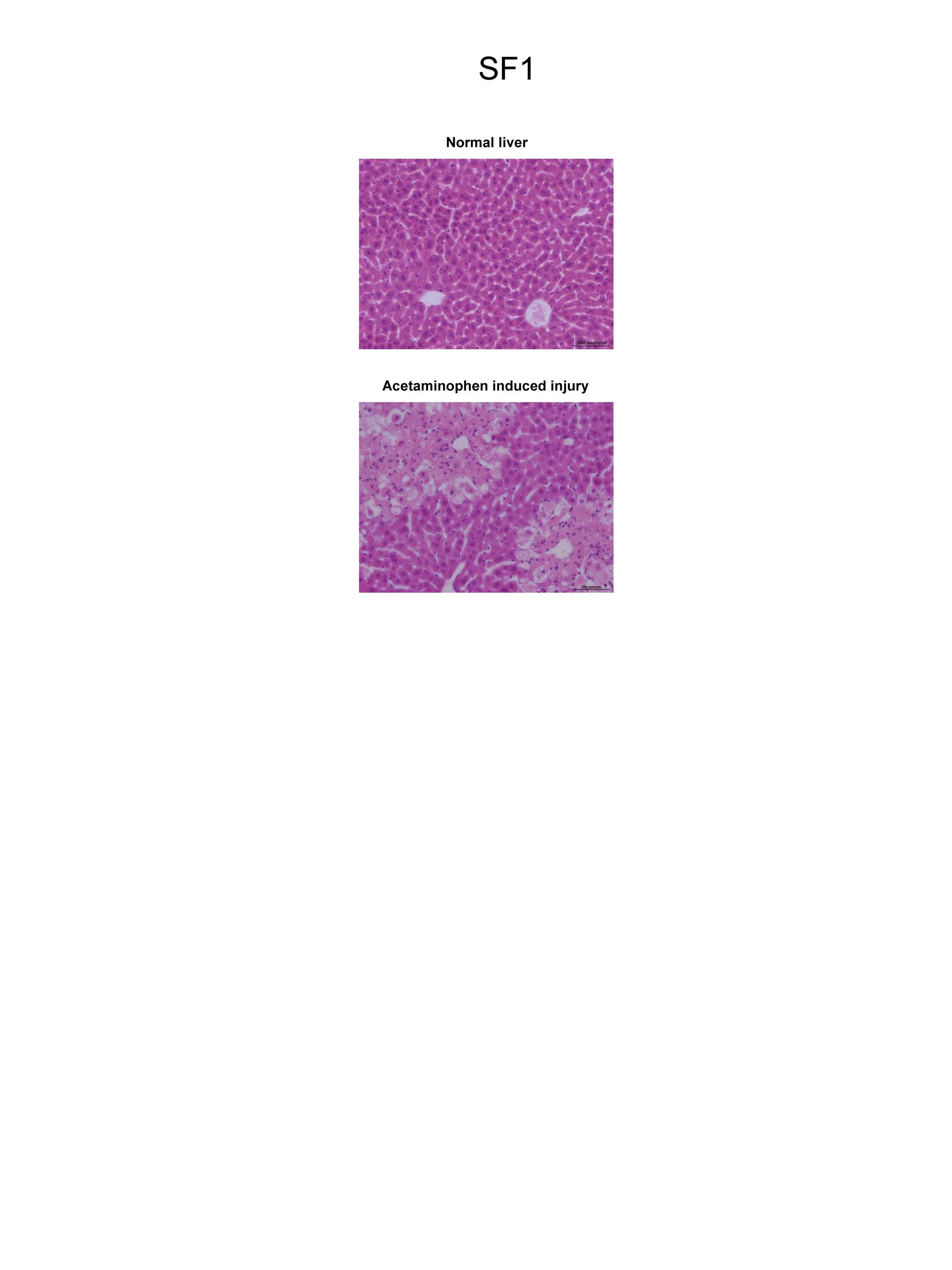

Supplement: Additional file 2: Figure S1. — Histopathology of liver sections after 24 h of acetaminophen-induced injury. (TIFF 1189 kb) [file 13287_2015_119_MOESM2_ESM.tiff]

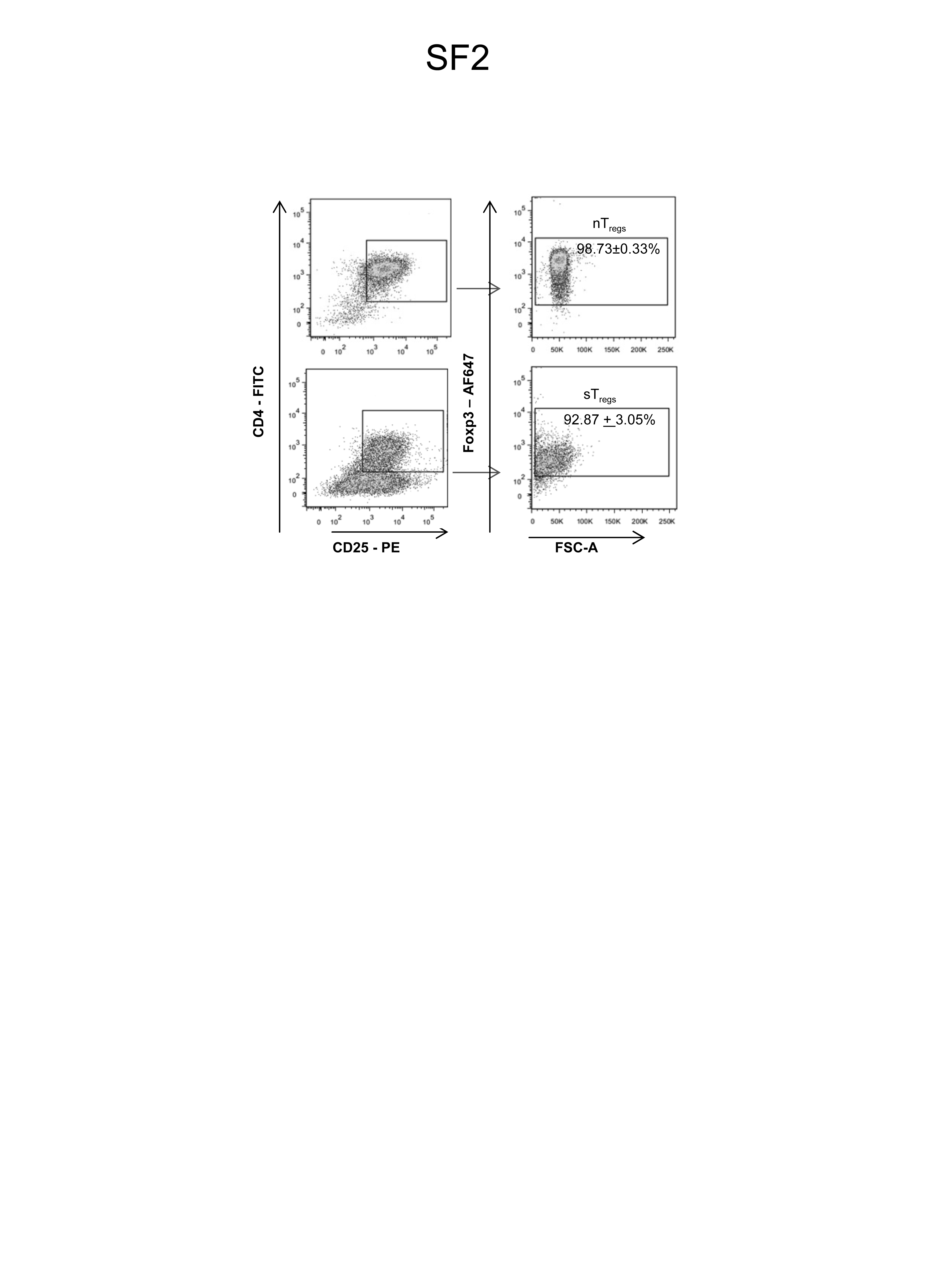

Supplement: Additional file 3: Figure S2. — Expression of Foxp3 in naïve (nTreg) and allo-sensitized (sTreg) regulatory T cells. (TIFF 523 kb) [file 13287_2015_119_MOESM3_ESM.tiff]

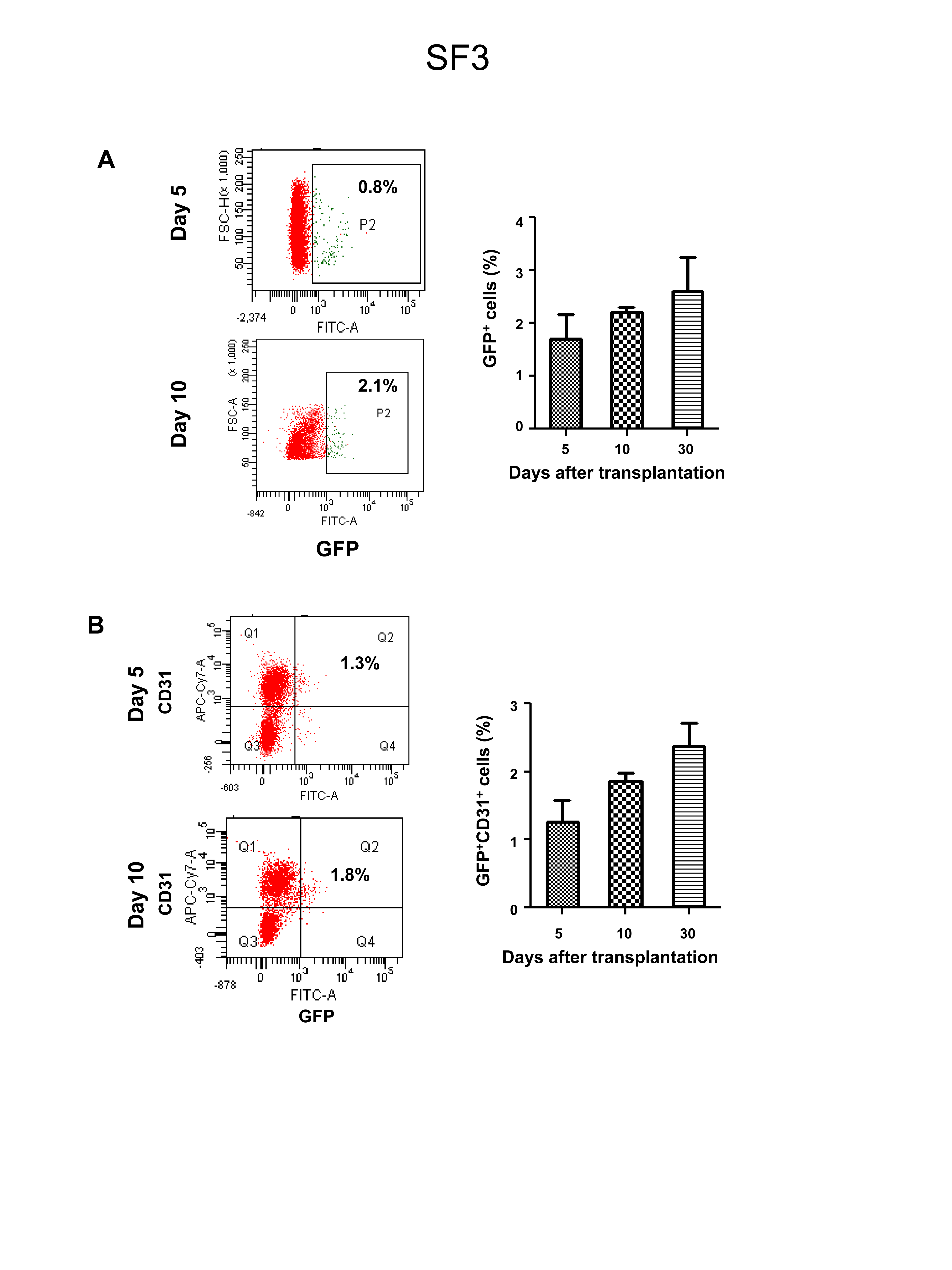

Supplement: Additional file 4: Figure S3. — Donor-derived non-parenchymal cells in HAT-AT liver. a Time course analysis of GFP+ donor cells in non-parenchymal cells of HAT-AT mice. b Time course analysis of GFP+ donor-derived endothelial cells in non-parenchymal cells of HAT-AT mice. GFP green fluorescence protein, HAT-AT hemophilia A mice transplanted with allogeneic and regulatory T cells. (TIFF 643 kb) [file 13287_2015_119_MOESM4_ESM.tiff]

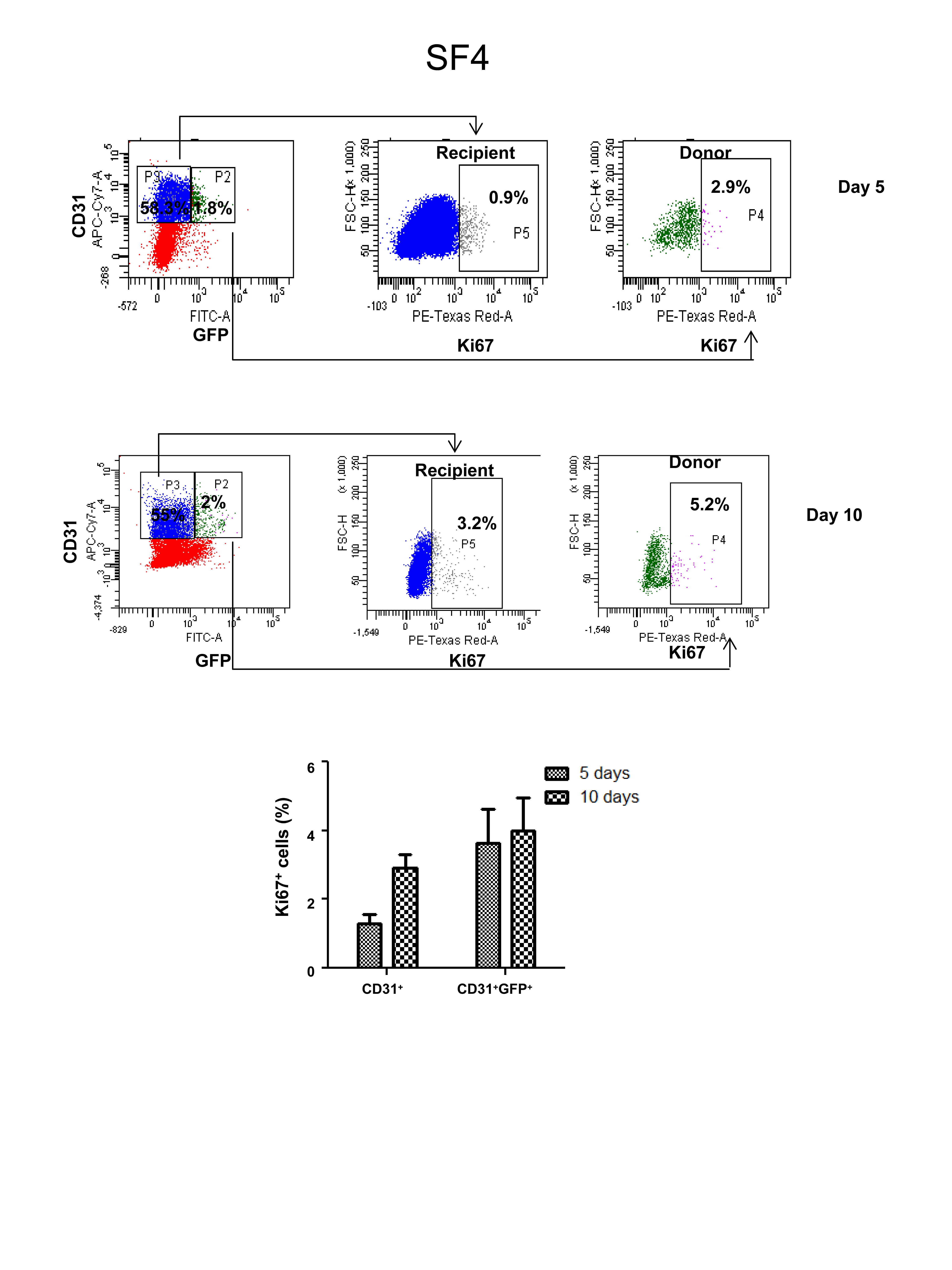

Supplement: Additional file 5: Figure S4. — Proliferation of endothelial cells in HAT-AT liver after 5 and 10 days of transplantation. Proliferating cells (Ki67+) were found to be present in both host and donor-derived fractions of endothelial cells. HAT-AT hemophilia A mice transplanted with allogeneic and regulatory T cells. (TIFF 733 kb) [file 13287_2015_119_MOESM5_ESM.tiff]

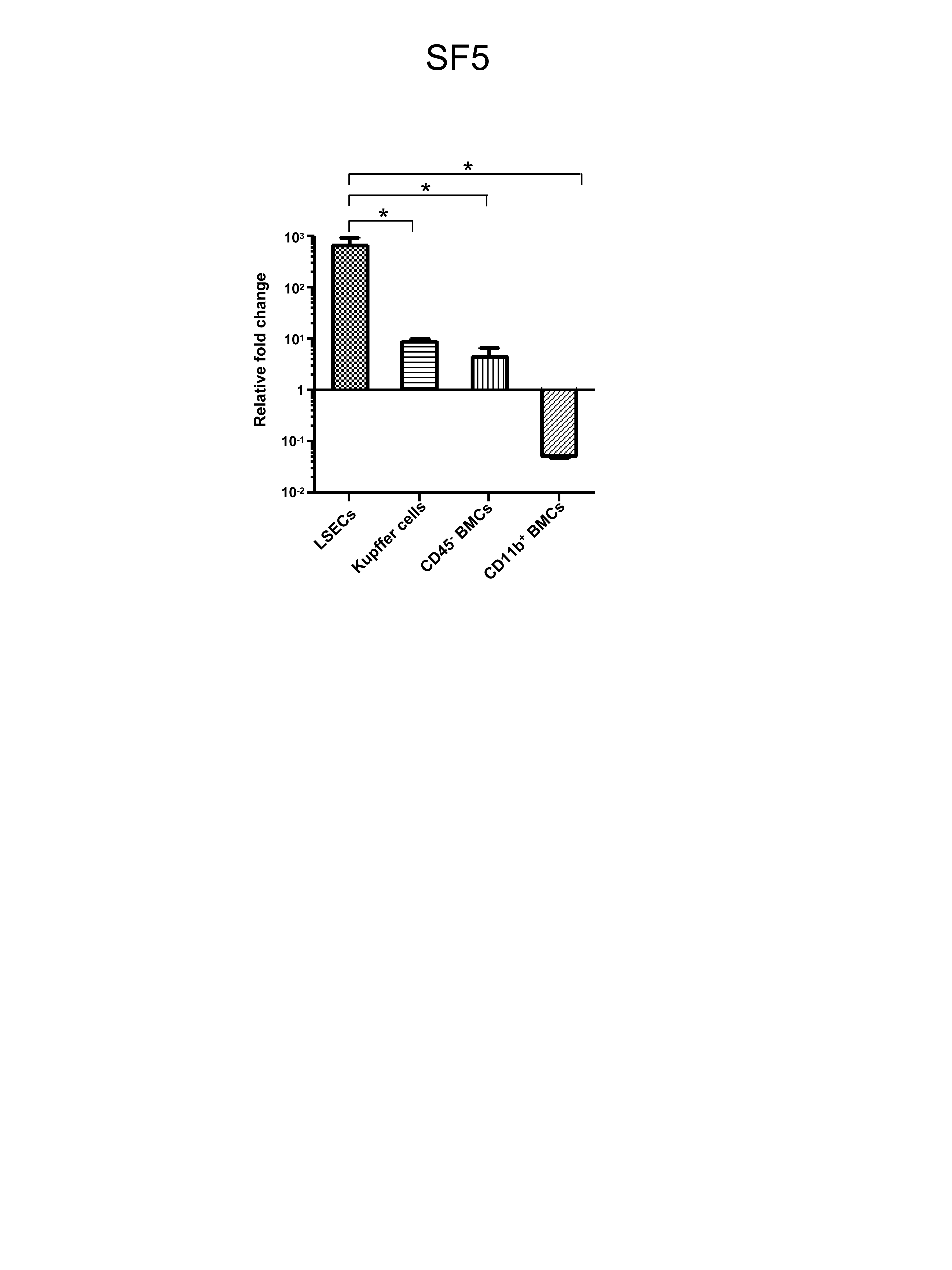

Supplement: Additional file 6: Figure S5. — Expression of FVIII mRNA in liver and bone marrow cells of wild-type mice. Fold change calculated with respect to expression in Lin− BMCs. BMC bone marrow cell, FVIII factor VIII, LSEC liver sinusoidal endothelial cell. (*) P < 0.05. (TIFF 183 kb) [file 13287_2015_119_MOESM6_ESM.tiff]

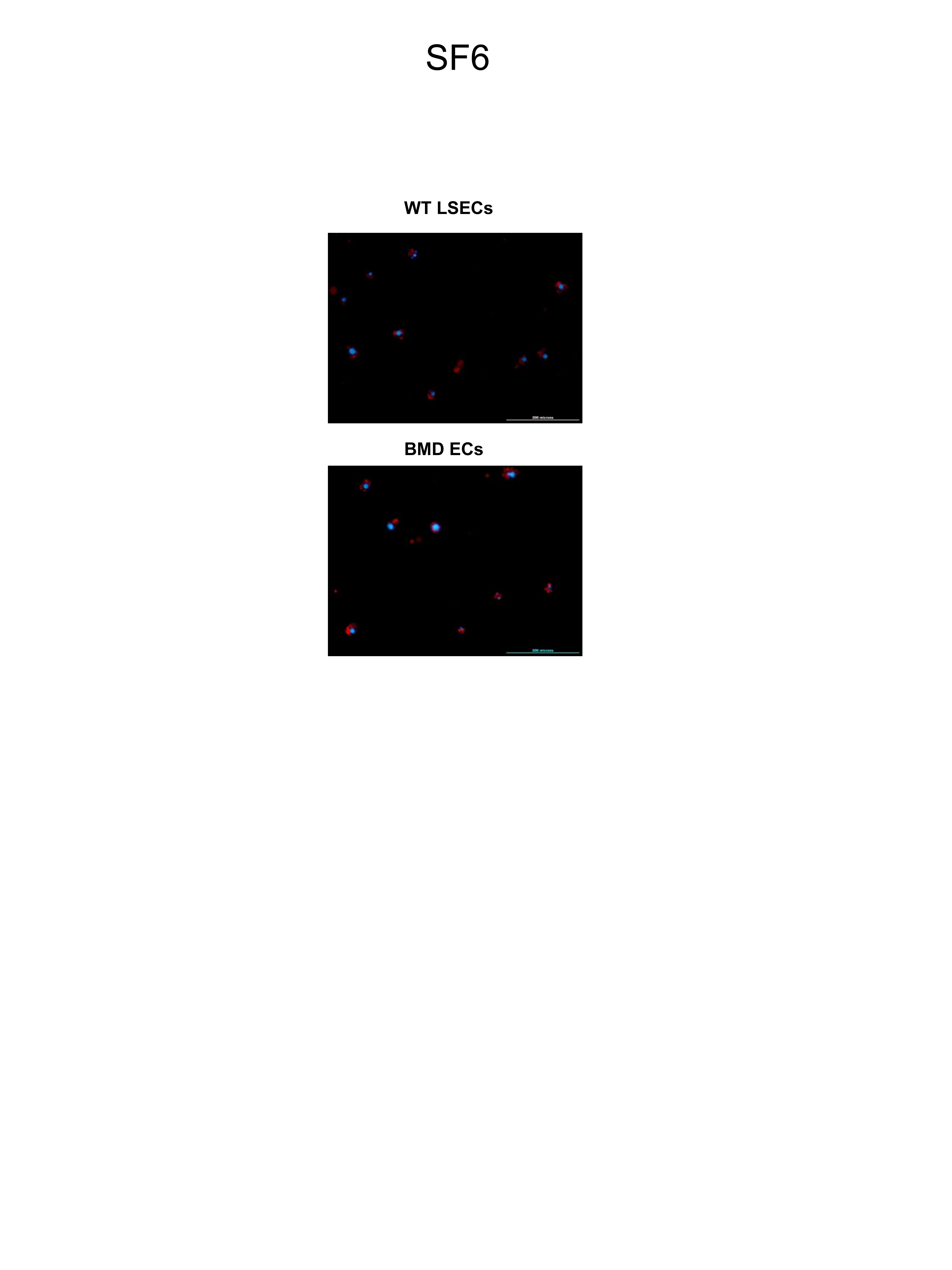

Supplement: Additional file 7: Figure S6. — DiI-Ac-LDL (*) P < 0.05 uptake in WT LSECs and donor BMD ECs from HAT-AT liver. BMD-EC bone marrow-derived endothelial cell, DiI-Ac-LDL 1,19-dioctadecyl-3,3,39,39-tetramethylindo-carbocyanine perchlorate, HAT-AT hemophilia A mice transplanted with allogeneic and regulatory T cells, WT LSEC wild-type liver sinusoidal endothelial cell. (TIFF 491 kb) [file 13287_2015_119_MOESM7_ESM.tiff]
